# Understanding discrepancies in parent-child reporting of emotional and behavioural problems: Effects of relational and socio-demographic factors

**DOI:** 10.1186/1471-244X-10-56

**Published:** 2010-07-16

**Authors:** Betty Van Roy, Berit Groholt, Sonja Heyerdahl, Jocelyne Clench-Aas

**Affiliations:** 1 University of Oslo, Institute of Psychiatry, Norway; 2 Akershus University Hospital, Division of Mental Health, Lorenskog, Norway; 3 Centre for Child and Adolescent Mental Health, Eastern and Southern Norway, Oslo, Norway; 4 Norwegian Institute of Public Health, Division of Mental Health, Oslo, Norway

## Abstract

**Background:**

Discrepancies between parents and children in their assessment of children's mental health affect the evaluation of need for services and must be taken seriously. This article presents the differences between parents' and children's reports of the children's symptoms and social impairment, based on the results of the Strengths and Difficulties Questionnaire (SDQ). The interrelationship between relational aspects and socio-demographic factors with patterns of disagreement are explored.

**Methods:**

Differences in the prevalence and means of SDQ symptom and impact scores were obtained from 8,154 primary school children, aged between 10 and 13 years, and their parents. Agreement between matched pairs was measured using Pearson's and Spearman's rho correlations. Socio-demographic variables, communication patterns and parental engagement were analysed as possible correlates of informant discrepancies using bivariate and multivariate logistic regression models.

**Results:**

In general, although children reported more symptoms, they reported less impact of perceived difficulties than parents. The parents were more consistent in their evaluation of symptoms and impact than were the children. Exploration of highly discrepant subgroups showed that, when children reported the most symptoms and impact, qualitative aspects of the parent-child relationship and family structure seemed to be more powerful predictors of disagreement than were gender of the child and socio-demographic variables. When parents reported the most symptoms and impact, low parental educational level, low income and male gender of the child played an additional role.

**Conclusions:**

Our findings underline the importance of paying attention to child reports of emotional-behavioural difficulties, particularly when parents do not identify these problems. Considerations on what meaning parent-child discrepancy might have in the context of the parent-child relationship or the family's psychosocial status should be integrated in the overall understanding of the child's situation and subsequent recommendations.

## Background

The multi-informant approach to the evaluation of children's mental health is widely recognized. However, only low to moderate agreement between informants has been found [1-4]. A meta-analytic review of 119 multi-informant studies by Achenbach et al. [1] showed that the mean Pearson's *r *between all types of informants was statistically significant, with a mean parent-child correlation of 0.25.

The Strengths and Difficulties Questionnaire (SDQ) is a standardised instrument to measure psychological adjustment among children and adolescents by measuring both emotional and behavioural symptoms and their impact on daily life. Parent, teacher and self-report use the same items and scales. Parent-child correlations from the SDQ, as reported in Goodman's study of 3,983 11-15-year-olds [5], were 0.48 for the total difficulties score and 0.30 for the impact scale. For the different subscales, the cross-informant correlations varied from 0.30 to 0.44. Other studies have also indicated that the SDQ correlations exceed the Achenbach meta-analytic mean [6-8].

A low to moderate agreement between parents and children on the SDQ, was recently highlighted in a clinical study of 11-18 year olds where 69% agreed that the children's problems were either clinically significant or not [9].

Limited parent-child agreement does not necessarily reflect lack of valid judgements by one informant, but can be due to the report of uniquely different information [10].

Research has shown that factors other than situational specificity may contribute to parent-child disagreement. Studies have focused on the severity and types of problems [1,4,9,11-14] clinical versus non-clinical populations [1,4,12,14] cultural and socio-economic aspects [12,15] and informant characteristics e.g. parental psychopathology [16,17] and children's age and gender [3,4,9,11,12,14,16,18]. The findings have been inconsistent and do not provide adequate conclusions [19].

Considerably less attention is given to the effect of family characteristics and relational aspects on parent/child agreement. Jensen et al. [20] found that families with a stepfather or adoptive father were associated with increased discrepancies between parents and children, compared with families with both biological parents.

Relational aspects and communication patterns between parents and children might influence the way parents perceive their children's behaviour and emotions. Treutler and Epkins [21] concluded that both qualitative and quantitative aspects of the parent-child relationship were related to discrepancies between parents and children. As Kolko and Kazdin note [12] they found that acceptance or rejection of the child played an important role in clarifying parents' ratings of their children's behaviour. In addition to time spent with children, the number of topics discussed between parents and children was inversely related to the discrepancies between informant's reports. Bidault-Russell et al. [22] showed that poor communication between adolescents and parents influenced their agreement.

Should qualitative aspects of the parent-child relationship -influence agreement on symptoms and impact, this knowledge should be integrated into the overall understanding of reporting differences and subsequent treatment recommendations.

This study describes differences and agreement on the various SDQ domains of child behaviour between more than 8,000 primary school children and their parents. Discrepancies both in reporting symptoms and in reporting their impact on daily life are explored. The possible effect of relational and socio-demographic factors on parent-child discrepancies was investigated.

We hypothesized that the SDQ results would confirm low to moderate agreement between parents and children's reporting of children's symptoms and impact. Further, we expected to find an interrelationship between different types of problems and parent-child discrepancies but, given the inconsistent findings in earlier studies, we had no clear hypothesis on how these factors would contribute.

Finally, we expected that relational factors in addition to socio-demographic factors were important contributors to parental-child reporting differences both for symptoms and impact.

## Methods

### Subjects

As part of a large epidemiological county study [23] both parents and their children completed a health profile questionnaire (5th-7th grade; 10 -13 years olds, mean age: 11.5; boys: 50%, girls: 50%). Classes at each school level were selected at random to obtain a sample representative of the county as a whole. Participation in the study was voluntary. The parents were informed by the local school and asked to give their consent. The children completed the questionnaire at school during regular classes under the supervision of the teacher (response rate: 87%), while the parents received the questionnaire at home via the child and returned materials in a sealed envelope (response rate 78%). There was no information concerning whom (mother, father or both) had filled out the questionnaire. The questionnaires of each parent and their child had the same registration number, so that they could be matched without violating the anonymity of the participants. As part of the questionnaire, 8,534 parents and 8,214 children filled out the SDQ (*N *= 8,154 matched cases; 73.1% of all preadolescents).

The study was conducted after approval from the Regional Committee for Medical and Health Research Ethics.

### Measures

#### Strengths and Difficulties Questionnaire (SDQ)

The SDQ (25 items), a brief questionnaire developed by Goodman [5] contains five subscales, each with five items covering emotional, conduct, hyperactivity and peer problems and prosocial behaviour. A total difficulties score (0-40) is generated by adding the subscale scores (0-10), except for the prosocial behaviour score. Norwegian cut-off scores were used to categorise the population into a high-risk group who scored above the 90th percentile (10%), a borderline group (10%) and a normal or low-risk group who scored below the 80th percentile (80%) [23].

The extended version of the SDQ includes a brief impact supplement. The respondent is asked whether he thinks that he/she/the child perceives any problems and, if so, is questioned further about chronicity, overall distress, social impairment related to family, friends, learning situations and leisure activities, and whether he/she is a burden to others. The five items concerning overall distress and social impairment generate an impact score ranging between 0 and 10. A total impact score ≥2 is defined as abnormal [5]. A score of 1 is defined as borderline. Those who answer "no" to the question of perceived difficulties get automatically an impact score of zero.

Similar versions of the SDQ can be completed by parents and by children aged from 11 to 16 years. Cross-cultural research has shown sound psychometric properties despite modest levels of internal reliability for several subscales [5-7,24].

#### Other relevant items

Information about socio-demographic characteristics and relational topics in the health questionnaires was used to analyse the factors of interest that might predict discrepancies between parents' and children's reporting of the children's mental health. The parents reported their highest education level (three levels: elementary school, high school and college/university), family income (less than €25,000 to more than €125,000), parental status (whether or not the child lived with both biological parents) and whether or not both parents were Norwegian.

The children's questionnaire explored parental engagement by the question "someone at home cares about what I do", the four response alternatives being yes, a little, no and don't know. Communication patterns were assessed by asking the children with whom they spoke most often: when they were happy, when they were sad and in their general mood. For each of these three questions, they could mark three different persons. For the logistic regression, a total communication variable was computed separately for mother, father, teacher, friends and others. This variable showed how often the child chose this person (e.g., the mother) from the response alternatives for the three communication questions (range 0-3). Parenting and communication issues were not addressed in the parents' questionnaire.

### Statistical methods

All analyses were performed using SPSS version 16.0. Differences in prevalence were tested using Pearson's Chi-square test. Differences in means were analysed with independent and paired-sample *t *tests and Cohen's *D *effect sizes (ES) for significant group differences, using pooled standard deviations.

Agreement on symptom scores was measured using Cohen's kappa and Pearson correlations. Spearman's rho correlation coefficient was used for total impact scores. *Z *test scores were calculated for significance testing of the differences between independent correlations. Pearson's correlations were used instead for intra-class correlations to have the opportunity to compare our results with other relevant studies on SDQ properties [5-7,25] and the meta-analytic mean in the study of Achenbach et al. [1].

Bivariate and multivariate logistic regression analyses were performed to explore possible predictors of disagreement between parents and children, expressed as odds ratios (OR) with 95% confidence intervals. Disagreement as an outcome variable was based on the comparison of a subgroup of parents who reported more symptoms than their children (parents' total difficulties score > 90th percentile and children's total difficulties score < 80th percentile), with a subgroup of parents and children who described equivalent levels of symptoms (the reference group). A similar comparison was conducted for a subgroup of children who reported more symptoms than their parents (children's total difficulties score > P90 and parents' total difficulties score < P80). In the same way, we compared the subgroup in which either parents or children reported an impact score, either ≥2 or equal to 0, with the subgroup reporting equivalent levels of impact. The exploratory variables school grade/age and communication were used as continuous variables. Income and education level were recoded as semi-continuous variables (OR expressed the differences in risk when income decreased by approximately €12.500 and when education level decreased by one year of education). The other variables (gender, parental status, parents' nationality and parental engagement) were used as categorical variables.

## Results

### Parents' SDQ and children's SDQ self-report scores

Table 1 row a) presents the means (SD) of the parents' and children's SDQ scores for boys and girls.

**Table 1:** Differences and agreement in SDQ ratings for reports from parents and self-reports

	Total difficulties			Emotional			Conduct			Hyperactivity			Peer problems			Prosocial			Impact		
	**mean (SD)**		***t-test.***	**mean (SD)**		***t-test.***	**mean (SD)**		***t-test.***	**mean (SD)**		***t-test.***	**mean (SD)**		***t-test.***	**mean (SD)**		***t-test.***	**mean (SD)**		***t-test.***
***a) MEAN (SD)***	**boys**	**girls**		**boys**	**girls**		**boys**	**girls**		**boys**	**girls**		**boys**	**girls**		**boys**	**girls**		**boys**	**girls**	

Parents' report N = 4,279/4,238	6.6 (5.2)	5.7 (4.8)	***	1.2 (1.7)	1.4 (1.8)	***	1.1 (1.4)	1.0 (1.2)	***	3.0 (2.4)	2.2 (2.0)	***	1.3 (1.7)	1.1 (1.6)	***	8.0 (1.7)	8.5 (1.5)	***	0.5 (1.3)	0.3 (1.0)	***

Self-report N = 4,101/4,096	10.1 (5.2)	10.0 (5.1)	n.s.	2.2 (1.9)	3.0 (2.2)	***	2.1 (1.7)	1.7 (1.4)	***	3.8 (2.1)	3.5 (2.0)	***	2.1 (1.8	1.9 (1.7)	***	7.4 (1.8)	8.2 (1.6)	***	0.3 (0.9)	0.3 1.0)	**

***b) PAIRED SAMPLE T-Test (parent/self-report)***	***	***		***	***		***	***		***	***		***	***		***	***		***	**	
*Effect size*	-0.67	-0.87		-0.56	0.80		-0.64	-0.54		-0.35	-0.65		-0.46	-0.48		0.34	0.19		0.18	0.00	

***c) CORRELATIONS ****Parent-child*			*z test*			*z test*			*z test*			*z test*			*z test*			*z test*			*z test*
Boys: 4,076/girls: 4,061	0.38**	0.40**	n.s.	0.25**	0.34**	***	0.27**	0.26	n.s.	0.33**	0.31**	n.s.	0.35**	0.33**	n.s.	0.23**	0.20**	n.s.	0.19	0.21	***

Total sample: N: 8,154	0.39**			0.30**			0.27**			0.33**			0.34**			0.24**			0.19**		

* *p *< .05, ** *p *< .01, *** *p *< .001; n.s. = non-significant

Independent sample t-test and effect sizes for gender differences; paired sample t-tests for differences in means between 8,154 matched cases.

Z-test: Significance of differences in Pearson's correlations between parents and sons, and between parents and daughters.

Impact: total impact score includes those who answered "no" to the question of perceived difficulties and automatically had an impact score of zero

#### Symptom reports by parents and children

For both genders, the mean total difficulties score was greater for children than for parents (*p *< .001). Boys reported significantly more problems than girls in all areas except emotional problems (ES: 0.12-0.49). The same pattern was observed in the parents' reports (ES: 0.08-0.36).

#### Impact reports by parents and children

Parents of boys reported more impact than parents of girls (p < .001) while self-report showed the same level of impact for both genders.

#### Associations between total difficulties scores and impact scores for parents and children (not reported in Table 1

Parents seemed more consistent in their evaluation of symptoms and impact than were their children. Of those parents who reported a total difficulties score > P90, 48.5% reported an impact score of ≥2, compared with 2.0% of the parents who reported total symptoms < P90 (OR: 45.1, 95% CI: 36.1-56.2). Of those children who reported a total difficulties score > P90, 26.9% reported an impact score of ≥2 compared with 2.4% of children who reported a total difficulties score < P90 (OR: 15.1, 95% CI: 11.7-19.4).

### Agreement between parents and children

Agreement was studied both by cross-informant correlations, by comparing means and by cross-tabulation of the high-risk, borderline and low-risk groups. While the correlations indicate similarities between the rank orders of scores assigned to the children by the informants, the mean differences and cross-tabulation yield information about the pattern of findings (e.g. which informant is reporting fewer or greater problems) [25].

#### Cross-informant correlations

The correlation between the total difficulties scores in parents' and self-report SDQ was 0.39 (*p *< .01) (Table 1 row c). At the subscale level, the correlations varied from 0.24 (prosocial behaviour) to 0.34 (peer problems). No gender differences were found, except for emotional problems (girls > boys, *p *< .001). The correlation coefficient for the total impact scores was 0.19, lower than for the total difficulties scores.

#### Differences in means

The paired sample t-test showed that children reported more symptoms than their parents but lower impact (Table 1 row b). For all the symptom scales, the differences in parent-child means were highly significant (*p *< .001) for both genders with moderate to high effect sizes (0.34-0.87). For the impact scale the difference in means (SD) between parents and boys was highly significant (p < 0.001), less significant between parents and girls (p < 0.01). The ES of the impact differences between parents and children was low for both genders (0.18 for boys and 0.00 for girls).

#### Cross-tabulation of the total difficulties and impact subgroups

After banding the total difficulties scores and impact scores into low-risk, borderline and high-risk groups, the majority of parents and children were located in equivalent groups, showing a modest overall agreement on both symptoms and impact (total difficulties: 78%, impact: 80%) (Table 2). A highly discrepant result for the total difficulties score was found in 8.6%, 5.3% involving parents who scored > P90 and children < P80 and 3.3% involving children who had a high-risk score and parents who had a low-risk score (*p *< .001; kappa: girls = 0.22, boys = 0.19). Of all the impact responses, 10.6% showed a highly discrepant result: 4.7% of cases in which children rated an impact score ≥2 and the parents reported an impact scale of 0, and 5.9% involving parents who obtained an impact score ≥2 and children reporting an impact scale of 0 (kappa = 0.14; boys = 0.14, girls = 0.15; a non-significant gender difference).

**Table 2:** Percentage agreement between parents and children (boys/girls) in total difficulties scores (*N*: 4,101/4,096) and in total impact scores (*N*: 4,007/4,011)

		Total difficulties score parents (boys/girls)						Total %	
		**Low risk** **< Percentile 80**		**Borderline** **Percentiles 80-90**		**High risk** **> Percentile 90**			

**Total difficulties score children**	Low risk: < P80	74.6	(72.4/76.9)^	5.4	(6.1/4.7)	**5.3**	(6.6/3.9)^^	85.3	(85.1/85.4)
	
	Borderline: P80-P90	5.5	(5.1/5.8)	1.3	(1.3/1.4)^	1.5	(1.8/1.3)	8.3	( 8.1/8.4)
	
	High risk: > P90	**3.3**	(3.5/3.2) ^^	1.0	(1.0/1.1)	2.1	(2.4/1.8)^	6.4	( 6.8/6.1)

Total %		83.4	(80.9/85.9)	7.8	(8.3/7.2)	8.8	(10.8/6.9)	100%	

		**Total Impact score for parents (boys/girls)**							

		**Low risk (score = 0)**		**Borderline (score = 1)**		**High risk (score ≥2)**		**Total %**	

**Impact score children**	Low risk ( = 0)	78.0	(76.3/79.7)^	3.5	(4.2/2.7)	**5.9**	(7.8/4.1)^^	87.4	(88.3/86.5)
	
	Borderline risk ( = 1)	4.2	(3.8/4.6)	0.5	(0.6/0.4)^	1.0	(1.2/0.7)	5.7	(5.6/5.8)
	
	High risk (≥2)	**4.7**	(3.9/5.4)^^	0.6	(0.5/0.7)	1.6	(1.6/1.6 )^	6.9	(6.1/7.7)

Total %		86.9	(84.0/89.8)	4.6	(5.3/3.8)	8.6	(10.7/6.4)	100%	

*p *< .001; ^ parents and children in equivalent groups; ^^ parents and children in highly discrepant groups

### The effects of socio-demographic and relational variables on discrepancies between parents' and children's SDQ scores

Data for the subgroups with highly discrepant total difficulties and impact scores were analysed further in order to define possible risk factors for the disagreement between parents and children (Table 3). The effects of gender, age, socio-demographic variables, communication patterns and parental engagement were studied in bivariate analyses before the simultaneous entry of all potential predictors (unadjusted OR with p < 0.5) in a multiple regression analysis (Table 4).

**Table 3:** Prevalence and means of different risk factors in subgroups of parent-proxy reports being respectively higher, equal or lower than child self-reports on symptoms and impact

	Risk factors in the three symptom subgroups Prevalence (%)			Risk factors in the three impact subgroups Prevalence (%)		
**Possible predictors**	**P > C** **N = 429**	**P = C** **N = 6,360**	**P < C** **N = 271**	**P > C** **N = 478**	**P = C** **N = 6,437**	**P < C** **N = 374**

**Gender**:						
Girls	36.8	51.1	48.0	34.4	51.0	58.0
Boys	63.2	48.1	52.0	65.6	49.0	42.0

**Educational level**:						
College/university	34.5	55.0	48.5	41.5	53.5	46.1
High school	53.4	39.0	43.3	48.2	40.3	47.7
Elementary school	12.1	5.9	8.1	10.3	6.2	6.2

**Parents' nationality**:						
Both Norwegian	84.2	86.5	91.1	84.9	86.5	86.6
Neither Norwegian	15.8	13.5	8.9	15.1	13.5	13.4

**Parental status**:						
Living with both	49.7	73.9	66.4	54.0	73.7	63.1
Not living with both	50.3	26.1	33.6	46.0	26.3	36.9

**Parental engagement**:						
Yes	69.2	80.9	57.0	72.1	80.6	61.5
Don't know	12.6	8.7	19.6	13.0	8.8	16.3
No	3.7	1.7	5.2	3.8	1.6	5.3
A little	14.5	8.7	18.1	11.1	9.0	16.6

	**Mean**			**Mean**		

**School grade/age **:mean age						
	11.5*	11.5*	11.4 *	11.4 *	11.5*	11.5 *

**Income**						
( n.100,000 crowns) mean income	412	494	478	430	491	449
*( n.100,000 *€ *)*	*(52 )*	*(62)*	*(60)*	(*54)*	*(61)*	*(56)*

**Communication**						
( range 0-3)						
Mother	2.5	2.6	2.5	2.6	2.6	2.4
Father	1.8	2.0	1.8	1.8	2.0	1.6
Teacher	0.2	0.1	0.2	0.2	0.1	0.2
Friends	1.6	2.0	1.8	1.6	1.9	1.9
Others (uncle, neighbour...)	0.3	0.1	0.3	0.2	1.5	0.3

P = parents; C = Children; Symptom subgroups based on total difficulties scores

**Table 4:** Effects of risk factors on disagreement in symptom and impact scores between parents and their children

	**Parents reporting more symptoms/*impact *than their children (N = 429/*478*), compared with pairs where parents and children agreed (ref) (N = 6,360/*6,437****)*				Children reporting more symptoms/*impact *than their parents (N = 271/*374*), compared with pairs where parents and children agreed (ref) (N = 6,360/*6,437*)			
**Possible predictors**	**Univariate OR (95% CI)**		**Multivariate (N = 6,557/*6666) *OR(95% CI)**		**Univariate OR(95% CI)**		**Multivariate (N = 6,415/*6,565*) OR( 95% CI)**	
	
	**Symptoms**	***Impact***	**Symptoms**	***Impact***	**Symptoms**	***Impact***	**Symptoms**	***Impact***

**Gender**: Boys	1.8 (1.5-2.2)***	2.0 (1.6-2.4)**	1.7 (1.3-2.1)***	1.9 (1.5-2.3)***	1.1 (0.9-1.4)	0.8 (0.6-0.9)**	1.0 (0.8-1.3)	*0.9 (0.7-1.0)*

**School grade/age**	1.0 (0.8-1.1)	*1.0 (0.9-1.1)*			1.0 (0.8-1.1)	*1.0 (0.9-1.2)*		

**Low Income**	1.2 (1.2-1.3)***	1.2 (1.1-1.2)***	1.1 (1.0-1.1)**	1.1 (1.0-1.1)*	1.0 (1.0-1.1)	1.1 (1.0-1.2)***		*1.0 (1.0-1.1)*

**Low Educational level**:	1.2 (1.1-1.2)***	1.1 (1.1-1.1)***	1.1 (1.1-1.2)***	1.1 (1.0-1.1)**	1.0 (1.0-1.1)*	1.0 (1.0-1.1)*	1.0 (1.0-1.1)*	*1.0 (1.0-1.0)*

**Parents' nationality **Neither Norwegian	1.2 (0.9-1.6)	*1.1 (0.9-1.5)*			0.6 (0.4-1.0)*	*1.0 (0.7-1.3)*	0.6 (0.4-1.0)*	*0.9 (0.6-1.2)*

**Parental status**: Not living with both	2.9 (2.4-3.5)***	2.4 (2.0-2.9)***	2.2 (1.8-2.8)***	2.0 (1.6-2.5)***	1.4 (1.1-1.9)**	1.6 (1.3-2.0)***	1.3 (1.0-1.7)	1.3 (1.0-1.7)*

**Parental engagement**:								
Yes (ref)	1.0		1.0		1.0		1.0	
	
Don't know	1.7 (1.3-2.3)***	1.7 (1.2-2.2)***	1.4 (1.0-1.9)	*1.3 (1.0-1.8)*	3.2 (2.3-4.4)***	2.4 (1.8-3.3)***	2.9 (2.1-4.1)***	2.2 (1.6-3.0)***
	
No	2.6 (1.5-4.5)***	2.6 (1.6-4.4)***	2.1 (1.2-3.7)**	2.2 (1.3-3.7)**	4.4 (2.5-7.9)***	4.5 (2.8-7.4)***	3.7 (2.0-6.9)***	4.0 (2.4-6.7)***
	
A little	1.9 (1.4-2.6)***	1.4 (1.0-1,9)*	1.7 (1.3-2.3)***	*1.3 (1.0-1.8)*	2.9 (2.1-4.1)***	2.4 (1.8-3.3)***	2.8 (2.0-4.0)***	2.3 (1.7-3.1)***

**Communication**								
Mother	0.9 (0.8-1.0)	*1.0 (0.9-1.1)*			0.8 (0.7-0.9)**	0.8 (0.7-09)***	0.9 (0.8-1.1)	*0.9 (0.8-1.3)*
	
Father	0.9 (0.8-0.9)***	0.9 (0.8-1.0)**	0.9 (0.8-1.0)	0.9 (0.8-1.0)*	0.9 (0.8-1.0)*	0.7 (0.7-0.8)***	1.0 (0.9-1.1)	0.9 (0.8-0.9)**
	
Teacher	1.5 (1.2-1.8)***	1.3 (1.1-1.6)**	1.4 (1.1-1.7)**	*1.2 (0.9-1.4)*	1.5 (1.2-1.9)***	1.4 (1.2-1.8)***	1.5 (1.1-1.9)**	1.3 (1.0-1.6)*
	
Friends	0.7 (0.7-0.8)***	0.7 (0.7-0.8)***	0.8 (0.7-0.9)***	0.8 (0.7-0.9)***	0.8 (0.7-0.9)**	*1.0 (1.0-1.1)*	0.9 (0.7-1.0)*	*1.0 (0.9-1.1)*
	
Others	1.2 (1.2-1.7)***	1.4 (1.2-1.6)**	1.2 (1.0-1.4)	1.2 (1.0-1.4)*	1.6 (1.3-1.9)***	1.5 (1.3-1.8)***	1.4 (1.1-1.6)**	1.3 (1.1-1.5)**

*p < .05; **p < .01; ***p < .001; symptom subgroups based on total difficulties scores

#### The effects of individual variables on disagreement in terms of odds ratios from bivariate logistic regression analyses

Some of the risk factors increased the likelihood for parent-proxy reports being both higher and lower than child self-report, both on symptoms and impact. These variables were low educational level of the parents, not living with both parents, lack of parental engagement, less communication with the father or friends and more communication with the teacher or others. In addition, parents with low income and parents of boys would be likely to report more symptoms and impact than did their children. Having two Norwegian parents increased the likelihood that children would report more symptoms than their parents, while being a girl and having parents with a low income predicted that children would report a greater impact than their parents.

#### Multiple logistic regression analyses of all potential predictors of disagreement in symptom and impact scores

With few exceptions, all variables that predicted that *parents *would report more symptoms and impact than their children remained significant in the multivariate analyses (Table 4).

When *children *reported more symptoms than their parents, lack of parental engagement, having two Norwegian parents, less communication with friends and more communication with teachers and others increased the risk of belonging to the disagreement group. The child's gender, income, educational level of the parents and parental status had no significant effects. When children reported most impact, lack of parental engagement was the strongest predictor (OR: 4.0, 95% CI: 2.4-6.7). Also, not living with both parents and more communication with the teacher increased the risk of disagreement.

## Discussion

This study confirmed that parents and children provide different information about children's mental health. Discrepancies were found in both symptom and impact reports. When children reported more symptoms and impact than their parents, disagreement was strongly associated with poor parental engagement and not living with both parents, and slightly with less communication between parents and the child. When parents reported more symptoms and impact, low educational level of the parent, low income and male gender of the child, were additional predictors of disagreement.

### How do parents and children report children's mental health?

In this study, children reported more symptoms but less impact of their perceived difficulties than did their parents. These discrepancies might reflect that self-reports mostly provide information about the child's perception and tolerance of his or her behaviour and feelings, primarily at the time of the evaluation, whereas parents' reports, to a greater extent, reflects overall symptoms occurring over time. Children may be more sensitive to minor disturbances and report them, even if those disturbances are associated with less impact and are less visible for their parents.

Parents showed more consistency in their evaluation of symptoms and impact than the children. Children more often described serious symptoms without perceiving severe difficulties and serious impact. This might indicate that Norwegian children are highly expressive about how they feel and behave, but not all the symptoms they reported were perceived as being problematic enough to impair their daily lives.

Norwegian parents, on the other hand, seemed to have a high threshold for describing their children's behaviour as problematic. Cross-cultural studies using the CBCL/YRS showed that Scandinavian parents report fewer symptoms than parents from most other countries, while Scandinavian adolescents report more symptoms than youngsters from most other countries [15,26,27]. A study, comparing parental SDQ scores between a Norwegian and British population described under-reporting/under-recognition of children's emotional problems by Norwegian parents and teachers [28].

### Similarities and discrepancies

The cross-informant correlation for the total difficulties score (0.39) was higher than the meta-analytic mean of the Achenbach et al. [1] study (0.25), lower than the SDQ results reported by Goodman [5] (0.48) and van Widenfelt et al.[8] (0.47), but similar to the results of Becker et al.[6] (0.39) and Koskelainen et al.[7] (0.38). In addition, the impact correlation value (0.19) was lower in the current study than in Goodman's study [5] (0.25).

Goodman's study was based on a population of adolescents aged 11-16 years, while the current study included only preadolescents aged 10-13 years. This supports the findings of other studies, suggesting that in community samples, older children have a tendency to agree more with their parents [3]. The discrepancy between our results and those of Goodman might also be related to national characteristics, reported in other studies [15,26-28].

Although other SDQ studies have indicated higher agreement on externalising problems (hyperactivity and conduct subscales) than on the emotional subscale, this was not the case in the current study in which cross-informant correlations of conduct problems were lower than those of both hyperactivity and emotional problems. These findings support that, for both internalising and externalising disorders, characteristics of the behavioural items reported are more relevant in the prediction of parent-child agreement than type of disorder. Herjanic et al. [29] suggested that agreement is higher on questions concerning symptoms that are concrete, observable, severe and unambiguous, while Karver [10] reported that saliency to the parents, saliency to the child and observability/willingness to report were the most relevant determinants in the prediction of agreement (e.g. low agreement on the stealing item in the conduct subscale vs. higher agreement on the headaches item in the emotional subscale).

Poor agreement on conduct problems compared to the other SDQ subscales may also be related to the construct validity of the SDQ. Several studies have reported problematic low internal reliability of the conduct subscale, suggesting that the items in this subscale are not only reflecting conduct problems and that results measured in this subscale should be interpreted with caution [24].

### Associations between socio-demographic and relational factors and patterns of agreement and disagreement

In agreement with the findings of Jensen et al. [20] it seems easier for parents and children to agree about the children's psychological functioning when children live with both biological parents (Table 4). This finding supports the idea that mothers and fathers are sensitive to different aspects of the child's observed behaviour [13,16], and that two parents develop a more complete view of their child than one alone. It also supports that both parents are important informants in the assessment of the children's mental health [30]. However, independent of parental status, qualitative aspects of the parent-child relationship appeared to contribute more strongly to parent-child discrepancies than socio-demographic factors. In particular parental engagement but also good communication between parents and their children were important to increase agreement between parents and children, as reported by Treutler and Epkins [21]. Especially, communication with the father seems related to discrepancies in reporting on emotional-behavioural problems and their impact. This should be explored further. For children who disagreed with their parents, the teacher seemed to be an important alternative adult, probably owing to a lower level of parental engagement.

### Strengths and limitations of this study

This study describes parent-child agreement in a large representative population with 8,154 matched cases of children (10-13 years) and their parents. The focus on discrepancies in impact reports, which has scarcely been studied previously, in addition to differences in symptom reports, has contributed an additional perspective to previous research on agreement between parents and their children. Findings on the interrelationship between relational and socio-demographic aspects and parent-child discrepancies illuminate the value of a multidimensional assessment approach.

Although the sample size, the impact data and the diversity of predictors were the major advantages of this study, there were some limitations. Clinical validation of the results was lacking. Discrepancies between parents and children do not tell us whether or not their reports are valid or accurate, but other studies have shown that both informants are important to reduce the false negative group [6,9].

In analyses of possible factors associated with disagreement patterns, comparisons were made between groups with the same or different total difficulties scores and total impact scores, based on knowing that two informants could agree about the overall level of problems without necessarily agreeing about any of the constituent symptoms. Even though the rank correlations showed different levels of agreement on the different subscales, the total difficulties score showed a higher correlation than any of the subscales and, for this reason, could be used as a good overall measure of agreement between parents and their children.

The identity of the parental respondent (mother or father or both) was unknown. According to other studies [30,31], it can be assumed that different cross-informant results might be found on the different subscales between children and their mothers, or between children and their fathers. It would have strengthened the study if it had been possible to distinguish the fathers/versus mothers responses regarding both boys and girls.

In the health profile questionnaire, no questions were asked about the parents' psychological functioning. Even though parental functioning, primarily of the mother, received much attention in earlier studies [17], this variable was not included as a possible predictor in the present analyses.

The main focus of this study is on relational aspects and parent-child communication as possible predictors of agreement. The measures are brief and not thorough, based on few but relevant questions. The limited item set can weaken the reliability and validity of the assessment of these key constructs. Further research is needed to confirm the findings in our study.

## Conclusions

This study has confirmed that neither parental nor self-report data can substitute for each other in the assessment of children's mental health. Findings summarised in this article underline the importance of paying attention to child reports of emotional-behavioural difficulties, particularly when parents do not identify these problems. The findings remind us that children's behaviour does not exist in isolation from other characteristics of the child, such as gender, or from other aspects of the child's personal relationships and family life.

Lack of agreement between parents and children should lead not only to consideration of which informant is most objective or valid, but also to questions about what meaning these differences might have in the context of the parent-child relationship or the family's psychosocial status and eventually lead to further assessment of these dimensions of the child's life.

## Competing interests

The authors declare that they have no competing interests.

## Authors' contributions

BVR: conceived the study, drafted the manuscript. BG: contributed in all stages of the paper, from formulation of research questions to the final draft. SH: contributed in the planning of the study and the interpretation of the findings. JCA: prinicipal investigator for collecting the data and contributed to formulating the research questions. All authors read and approved the final manuscript.

## Pre-publication history

The pre-publication history for this paper can be accessed here:

http://www.biomedcentral.com/1471-244X/10/56/prepub
